# The Simple View of Reading in Children Acquiring a Regular Orthography (Italian): A Network Analysis Approach

**DOI:** 10.3389/fpsyg.2021.686914

**Published:** 2021-08-02

**Authors:** Paola Angelelli, Daniele Luigi Romano, Chiara Valeria Marinelli, Luigi Macchitella, Pierluigi Zoccolotti

**Affiliations:** ^1^Laboratory of Applied Psychology and Intervention, Department of History, Society and Human Studies, University of Salento, Lecce, Italy; ^2^Department of Psychology, Milan Center of Neuroscience (NeuroMi), University of Milano-Bicocca, Milan, Italy; ^3^Department of Clinical and Experimental Medicine, University of Foggia, Foggia, Italy; ^4^Center for Neurodegenerative Diseases and the Aging Brain, Department of Clinical Research in Neurology, Pia Fondazione Cardinale G. Panico, University of Bari Aldo Moro, Lecce, Italy; ^5^Department of Psychology, University of Roma “Sapienza”, Rome, Italy; ^6^Neuropsychology Unit, IRCCS Fondazione Santa Lucia, Rome, Italy

**Keywords:** transparent orthography, reading comprehension, reading accuracy, reading fluency, linguistic comprehension

## Abstract

In the present study, we explored the unique contribution of reading accuracy, reading fluency and linguistic comprehension within the frame of Simple View of Reading (SVR). The experimental sample included 118 3rd to 5th grade children learning Italian, a language with a highly regular orthography. We adopted a flexible method of analysis, i.e., the Network Analysis (NA), particularly suited for exploring relations among different domains and where the direct relations between a set of intercorrelated variables is the main interest. Results indicated an independent and unique contribution of syntactic comprehension skills as well as reading fluency and reading accuracy in the comprehension of a written text. The decoding measures were not directly associated with non-verbal reasoning and the latter was not directly associated with reading comprehension but was strongly related to oral syntactic comprehension. Overall, the pattern of findings is broadly consistent with the predictions of SVR and underscores how, in an orthographically regular language, reading fluency and reading accuracy as well as oral comprehension skills directly influence reading comprehension. Data are discussed in a cross-linguistic perspective. Implications for education and rehabilitation are also presented.

## Introduction

Reading comprehension is a multifaceted cognitive task that is critical for achieving good results in formal instruction, employment and the activities of daily living. In fact, a wide range of actions rely on individual ability to extract meaning from written texts. In this regard, it is important to frame individual performance within models of reading comprehension that are able to identify individual differences and may help improve both learning curricula and clinical interventions.

One influential model, i.e., the Simple View of Reading (SVR), proposes that processes which determine reading comprehension (R) are captured by two sets of skills: decoding (D) and linguistic comprehension (L) (Gough and Tunmer, 1986; Hoover and Gough, 1990). Decoding is defined as the ability to read isolated single words “*quickly, accurately and silently*” (Gough and Tunmer, 1986; page 7). Linguistic comprehension (L) is defined as the controllare originale:“*process by which, given lexical* (i.e., *word) information, sentence and discourses are interpreted*” (Gough and Tunmer, 1986, page 7). Thus, it refers to higher cognitive processes that go beyond reading and concern the oral language system.

The SVR also predicts that the influence of decoding and linguistic comprehension on reading comprehension will change as a function of schooling and reading proficiency. In the first years of formal instruction, children’s decoding skills, not linguistic comprehension, predict reading comprehension. New readers are primarily involved in the effortful task of phonological decoding and word recognition and only invest residual resources in reading comprehension. With reading experience, as children master decoding the relationship between decoding and reading comprehension decreases, and children’s differences in linguistic comprehension skills will become the most significant predictors of reading comprehension (Gough and Tunmer, 1986; Hoover and Gough, 1990).

The SVR model has been widely tested in readers of English, a highly opaque orthography, but also in readers of intermediate orthographies (such as French, i.e., Massonnié et al., 2019; European Portuguese, i.e., Cadime et al., 2017; Santos et al., 2020; see also Sparks and Patton, 2016 for Spanish L2 learners and Buil-Legaz et al., 2016 for Spanish-Catalan bilingual children with language deficits) as well as more transparent alphabetic scripts such as Finnish (e.g., Torppa et al., 2016), Greek (e.g., Protopapas et al., 2012, 2013; Kendeou et al., 2013) and Italian (e.g., Roch and Levorato, 2009; Tobia and Bonifacci, 2015). Evidence is also available for readers of some non-alphabetic writing systems, such as Chinese and Arabic (e.g., Joshi et al., 2012; Yeung et al., 2016; Asadi et al., 2017).

It is generally thought that reading decoding and linguistic comprehension explain a large part of the variance in reading comprehension in both orthographic and non-orthographic systems (for reviews see Florit and Cain, 2011; García and Cain, 2014). However, contrasting results have also been reported and some questions still remain. First, different patterns of associations among word decoding, linguistic comprehension and reading comprehension can emerge in different alphabetic orthographies and at different stages of reading because word reading is generally acquired much more easily in transparent orthographies (see for example the meta-analysis by Florit and Cain, 2011). Cross-linguistic studies have shown that orthographic consistency strongly influences the rate and modality of reading acquisition across different languages (for a review, see Ziegler and Goswami, 2005). Studies report longer time needed in opaque orthographies to master reading (e.g., Seymour et al., 2003) and a greater reliance on lexical procedure/larger print-to-sound units than in consistent orthographies (e.g., Ziegler et al., 2001). In the latter case, the fast rate of reading acquisition makes available greater cognitive resources for higher comprehension processes. Thus, readers of transparent orthographies may demonstrate a weaker relationship between decoding and reading comprehension and a stronger one between linguistic and reading comprehension in the early stages of reading acquisition with respect to learners of opaque orthographies (see for example Müller and Brady, 2001; but also see Zamperlin and Carretti, 2010; Tobia and Bonifacci, 2015).

Moreover, the relations between decoding and reading comprehension might depend on the way decoding is measured. In fact, the definition of word recognition as the ability to read single words “*quickly, accurately and silently*” (Gough and Tunmer, 1986; page 7) is somewhat underspecified and has been operationalized in several different ways. Most studies of English readers have measured only the decoding accuracy of either words or non-words. However, there is evidence that reading accuracy and reading rate are correlated but separable constructs (e.g., Bowers and Wolf, 1993; Carver, 1998). Bowers and Wolf (1993) discussed several lines of convergent research that point out precise time mechanisms which, if defective, can hamper efficient connections between phonological and orthographic codes and affect the quality of orthographic codes. Furthermore, accurate decoding may be insufficient to guarantee reading comprehension: if decoding is difficult and not automatized, the attention and cognitive resources necessary to process meaning will be insufficient, resulting in poor comprehension. Automaticity is considered an essential component of fluency^1^ (see for example Fuchs et al., 2001).

However, data on English readers are inconclusive with regard to whether decoding fluency adds to the prediction of reading comprehension beyond decoding accuracy: some authors find that it makes a significant contribution (e.g., Silverman et al., 2013), while others do not (e.g., Adolf et al., 2006). Also, for transparent orthographies the results are mixed. It is well-known that fluency^2^ intended as reading rate is a particularly sensitive marker of word recognition in transparent orthographies (e.g., Zoccolotti et al., 1999). However, the relative contribution of reading accuracy and fluency to reading comprehension has been rarely analyzed in transparent languages (Florit and Cain, 2011). This issue is particularly relevant for the Italian language, which is the topic of the present study. Italian is a very shallow language that is characterized by high consistency of grapheme-to-phoneme correspondence and a high degree of accuracy in reading both words and non-words by the end of first grade (e.g., Cossu et al., 1995; Orsolini et al., 2006).

Results for Italian are contrasting. In a first study, Florit et al. (2008) observed the strongest correlations between reading comprehension and reading speed in third grade and between reading comprehension and oral comprehension in fifth grade. In a subsequent study, Roch and Levorato (2009) tested the SVR in adolescents with Down’s Syndrome (mean age 15 years) and in a small control group of first graders matched for reading comprehension to the participants with Down’s Syndrome. Word reading fluency was the strongest predictor of reading comprehension in the control group, whereas non-word accuracy did not make a unique contribution to reading comprehension over and above listening comprehension. Also Zamperlin and Carretti (2010) and Tobia and Bonifacci (2015) analyzed the development of relations between reading fluency and oral and reading comprehension in children in different grades (from 1st to 8th grade in Zamperlin and Carretti, 2010; from 1st to 5th grade in Tobia and Bonifacci, 2015). In both studies, oral comprehension was a stronger predictor of reading comprehension than reading decoding measures at all grades. In Tobia and Bonifacci (2015) study, reading accuracy played a significant but minor role, and reading fluency was never significant. In Zamperlin and Carretti (2010) study reading fluency was no longer statistically significant in secondary school. Finally, a recent investigation by Florit et al. (2020) on Italian children reported partially inconsistent results. The study was conducted on first-grade children assessed in two different observational moments: at the beginning of the school year (no formal instruction) and after 6 months of schooling. Listening comprehension had a stronger relationship with reading comprehension than both reading decoding parameters; however, also reading fluency, not accuracy, significantly influenced reading comprehension although with a lower magnitude with respect to listening comprehension. In the model, vocabulary measures also played an important role in reading comprehension. In sum, studies of Italian students using the SVR model components present mixed results and the relative contribution of word recognition and linguistic comprehension to reading comprehension remains unclear. Furthermore, some studies reported a greater role of reading rate than accuracy while others failed to detect any role for reading fluency.

The present study aimed to further investigate the relationship between reading decoding (in terms of both fluency and accuracy), reading comprehension and listening comprehension in a sample of third- to fifth-grade Italian children, i.e., who are at a developmental stage in which instrumental decoding rules have been largely acquired. As we were interested in having a large spread of performance in all of these critical measures, we recruited a group of children who attended school regularly and excluded only children with very low non-verbal intelligence. To test the predictions of the SVR model, we relied on the Network Analysis (NA). This is a flexible method for exploring the relationships among various domains, where the main interest is the direct relationship between a set of variables that are intercorrelated with one another.

A network is a model that consists of a set of nodes, which represent entities, and a set of edges that connect the nodes, which represent their relations (e.g., De Nooy, 2011). NA is now used widely in the field of psychology due to its specific characteristics: (a) it is strongly data-driven (i.e., the final model is selected by adopting parameters derived from the data), (b) but can still be used to support theoretical hypotheses, (c) it allows analyzing complex sets of interrelated variables, (d) giving back reliable, parsimonious and replicable results, (e) where the researcher can look simultaneously at multiple variables that are at the same time predicted and predictors (Epskamp et al., 2017). The behavior can be explained as an emerging property of the observed pattern of relations between the variables (Costantini et al., 2015). With NA, the researcher does not need to have an *a priori* model, as in the case of confirmatory factor analysis, thus leaving any possible relationship free to emerge from the data. Additionally, the use of undirected relations allows considering circularity in the studied relations. In this investigation, we used NA to inform about the following questions: (a) the role of reading skills in the reading comprehension of Italian primary school children in the higher grades; (b) the relations between reading fluency, reading accuracy, linguistic comprehension, and reading comprehension, ruling out the contribution of a potentially relevant variable such as non-verbal intelligence. In particular, we examined the role of syntactic comprehension as well as accuracy and speed in reading a meaningful text in explaining text comprehension in a sample of third- to fifth-grade children. We chose an oral syntactic comprehension test because of the generally low variability shown by other tests (such as receptive vocabulary) at the age examined here. Reading skills were explored with the reading aloud of a meaningful passage, in order to have a more functional and ecologically valid measure. The inclusion of non-verbal intelligence as a node in the model allowed studying the unique quote of variance shared by reading decoding, linguistic comprehension and reading comprehension, partialling out the role of non-verbal reasoning.

## Materials and Methods

### Participants

A sample of 118 Italian children (50 females and 68 males), ranging in age from 7.9 to 11.2 years (average age: 9.80 ± 0.80), were recruited from three primary schools in southern Italy. The only exclusion criterion was performance on Raven’s Colored Progressive Matrices (Raven, 1965) below the normative values (at least—2 SDs) based on Italian norms (Pruneti, 1996). In particular, 24 3rd grade children (average age: 8.55 ± 0.04), 41 4th grade children (average age: 9.51 ± 0.35) and 53 5th grade children (average age: 10.58 ± 0.30) participated in the study. All children attended school regularly, and none were singled out for socio-economic disadvantage by their teachers. The study was performed in schools in southern Italy, in areas without major migratory flows and devoted to primary and secondary economic sectors. The parents were informed about the research activities and authorized their child’s participation by furnishing written informed consent. The study was conducted according to the principles of the Helsinki Declaration and was approved by the school authorities and by the Ethics Committee of Psychological Research of the Department of History, Society and Human Studies—University of Salento (Prot. 101206 -29th July 2020).

### Materials

#### Non-verbal-Intelligence

Raven’s Colored Progressive Matrices is a non-verbal test of intelligence and reasoning (Raven, 1965). The test provides a simplified 36-item paper format. Each item contains a pattern problem with one part removed and six pictured inserts, one of which contains the correct pattern. Subjects must select the pattern that completes the target figure. No time limit was given for the task and the standard administration procedure was used. Correct responses were computed (maximum score = 36) and the score was transformed into a z score according to Italian normative data (Pruneti, 1996).

#### Text Reading Task

Participants’ reading level was assessed by administering a standard reading achievement test widely used for Italian children (MT reading test, Cornoldi and Colpo, 2011). The test consists of a series of meaningful texts (short stories taken from children’s books) that vary in length and complexity depending on the school grade (from grades 1 to 8) with related grade norms. The length of the text passages used in the present study varied from 168 words in grade 3 to 215 words in grade 5. Each story was printed in black on a white piece of cardboard. None of the texts used for this task were used for the text reading comprehension task (see below). Children read a single text depending on their grade; they were asked to read the text aloud as correctly and fluently as possible within a 4-minute time limit There was no reference to reading comprehension in the instructions. Two parameters were computed: (1) reading fluency obtained by the number of syllables read/seconds; (2) accuracy calculated as the number of errors, adjusted for the length of the text read. Following the manual, accuracy scoring takes into account the functional meaning of errors. Each word with an elision, substitution, insertion or inversion of letters is scored as 1 error, while changes in stress assignment, hesitations, spontaneous self-corrections, errors that do not change the meaning of the text and repetitions of the same errors are given a 1/2 score. Raw individual data were transformed into z scores, according to norms of their reference-grade groups (Cornoldi and Colpo, 2011). Each performance was recorded in order to check errors, also with an offline correction. Test-retest indexes for reading speed, as reported by the manual, ranged between 0.85 and 0.96.

#### Text Comprehension Task

The task materials consisted of a series of narrative texts (Cornoldi and Colpo, 2011). For 3rd to 5th grade, texts ranged in length from 226 to 306 words and their length increased with school grade (a different text was used for each grade). The children were asked to read the text in silence at their own pace; then, they had to respond to 10 multiple choice questions, choosing one out of four possible alternatives. The comprehension questions concern information that is either explicitly stated or implied by the text. There is no time limit and the children are allowed to return to the text while responding to the questions in order to minimize memory load. The final score is calculated as the total number of correct responses. Raw individual data were transformed into z scores, according to the norms of their reference groups (Cornoldi and Colpo, 2011). Alpha coefficients, as reported by the manual, ranges between 0.61 and 0.83 depending on grade.

#### Syntactic Comprehension Task

Syntactic comprehension was assessed by administering the Syntactic Comprehension Task (SC-T), which is a test adapted from the TROG by Bishop (1989) and which is part of the Child Neuropsychological Battery (Bisiacchi et al., 2005), a comprehensive battery of tests designed to assess various neuropsychological skills in children aged 5 through 11. The SC-T consists of 18 items: the child listens to a sentence and is asked to identify which, among four pictures (to choose which picture out of four alternatives is the one that represents the meaning of the sentence. The wrong alternative options include distractors related to the correct response. The distractors can be lexical (items 1–8) or syntactic alternatives (items 9–18). No time limit is given. One point is given for each correct response (maximum score = 18). The total accuracy score obtained by each child was transformed into a z score, in line with the normative data (Bisiacchi et al., 2005).

#### Procedure

The tests were administered in two sessions. The intelligence, text reading and syntactic comprehension tasks were given individually in a quiet room in the children’s school. The sequence of tests was randomized across participants. The individual session was ca. 35–45 min long. The MT comprehension test was group-administered and took about 10 min.

#### Statistical Analysis

Networks are a convenient option for modeling complex patterns of relationships. They allow analyzing several variables and the complexity of their relationships and give back readable outputs and indices. Networks are transdisciplinary and in psychology have been somewhat classical to model personality, psychopathology and attitudes (Cramer et al., 2012; Schmittmann et al., 2013; Costantini et al., 2015, 2017; Dalege et al., 2016; Borsboom, 2017). More recently, networks have been used to characterize neuropsychological performances in adults (Tosi et al., 2020; Ferguson and Alzheimer’s Disease Neuroimaging Initiative, 2021) and to understand the relationship among math, reading and spelling skills in children (Zoccolotti et al., 2021).

A network is composed of a set of elements named nodes (i.e., the variables) and their connections named edges (i.e., the relationship). In networks assessing psychological phenomena, the edges are typically estimated with the Gaussian Graphical Model (GGM; Epskamp et al., 2017). Within GGM, an edge expresses a regularized partial correlation.

In this study, a GGM network was estimated using the graphical “*least absolute shrinkage and selection operator*” (LASSO; Tibshirani, 1996) algorithm as a regularization parameter (Friedman et al., 2008). The value of the LASSO is chosen by using the Extended Bayesian Information Criterion, a method for carrying out quantitative model selection, which is tuned by a parameter γ and which we set at.25, as suggested in the literature (Epskamp, 2016). The adoption of the LASSO leads small connections to shrink to zero (McNeish, 2015; Epskamp and Fried, 2018). The scope of the LASSO is to return a conservative network model that reduces overfitting and limits false-positive edges, producing replicable and interpretable results (Costantini et al., 2015).

Thus, how should an edge be interpreted? Two nodes are conditionally dependent when an edge connects them. The connection can be read as a partial correlation, i.e., the association between the two variables, net of the variance explained by the other variables in the network. This means that the association cannot be explained by the fact that another association is part of the network and the relation between the two variables is direct. When two nodes are disconnected, it means that they are conditionally independent, given with respect to the other nodes of the network, i.e., there is no variance shared uniquely by the two variables.

GGM may have a low sensitivity (i.e., not all real edges are detected) but it has a high specificity (i.e., few false positives) because of the regularization parameter (Epskamp et al., 2018). It is important to note that the potentially missing edges are those that are smaller and less consistent. On the contrary, this method basically does not produce false positives; thus, if an edge is estimated, it has to be considered to be true (Epskamp et al., 2018).

GGMs can also be read as a predictive model. The neighbors of each node correspond to its predictors. A node with high strength centrality is also a node that is highly predictable given the others, quantifying the predictability of a node according to the number of its neighbors and the strength of its connections (Barrat et al., 2004).

Notably, the GGM relations are undirected. The consequence of adopting undirected relations is that circular homeostatic effects can be detected and interpreted (e.g., A influences B which influences C which influences A), while using directed methods, like structural equation modeling, circularity cannot be observed. Networks are not better *per se*, but they may provide a different perspective that can obtain converging evidence from different methodological approaches in independent studies. However, they offer the important feature of looking simultaneously at multiple variables that are predicted and predictors at the same time.

The stability of the results was checked using a bootstrapping procedure (1.000 resampling); the bootstrapping leads in calculating the confidence intervals (CI) of each edge. By inspecting CIs, one can identify different types of edges. The edges that do not include 0 are stronger and more likely to be replicated. For these edges, one can expect to find an edge different from 0 in 95% of the samplings; thus, it is likely to be replicated in future studies. The edges estimated as different from 0, but including 0 in the CI, highlight the associations that could pass undetected in different samples (e.g., in a replication). Notably, GGM adopting the eBIC Lasso estimator is known to be particularly reliable in not producing false-positive results; thus, one can interpret these edges as very likely to be true (i.e., as the first type presented). At the same time, one should also have careful consideration in expecting future replications. Finally, edges that have CI crossing the 0 are particularly unreliable because future replications are expected to also find results in the opposite direction. This class of edges should therefore be considered as 0.

JASP software (JASP Team, 2020) was used to run the analyses. JASP (Version 0.14)(Computer software) is a software that grounds the network module on the *bootnet* and *qgraph* (Epskamp et al., 2012) packages of the R statistical software (R Core Team, 2020).

## Results

Means, standard deviations and ranges for the variables of interest are reported in Table 1. Mean z-transformed data for measures of reading comprehension, reading speed/accuracy, and syntactic comprehension were close to zero, indicating marginal deviations from the same age standardization samples. Notably, a large spread of performance was present for all variables, indicating no clear evidence of a restriction of range or a ceiling effect. Also mean performance for the measure of non-verbal intelligence was close to zero; note that due to the exclusion criteria none of the children performed below 2 SDs on Raven’s test.

**TABLE 1:** Means, standard deviations and range values for Comprehension, Reading fluency, Reading accuracy, Syntactic comprehension, and Non-verbal intelligence.

Variable	Comprehension	Reading speed	Reading accuracy	Syntactic comprehension	Non-verbal intelligence
Mean	–0.24	–0.368	–0.378	0.229	–0.242
Standard deviation	0.758	0.809	1.091	0.845	0.8
Minimum	–2.72	–2.863	–4.702	–2.44	–1.91
Maximum	0.61	1.48	0.9	1.54	1.35

*Values indicate z scores with respect to standard norms.*

Inspection of individual data showed that 95% of participants had average or good levels of performance in reading comprehension (only six children underperformed), 97% in reading fluency, 94% in reading accuracy and 99% in syntactic comprehension. Overall, only nine children (7.6%) showed a marked reading delay (two children for both reading parameters, two only for reading fluency, and five children only for accuracy), and only one child underperformed in the syntactic comprehension task. They were not removed from the analyses because none had cognitive impairments.

**FIGURE 1:**
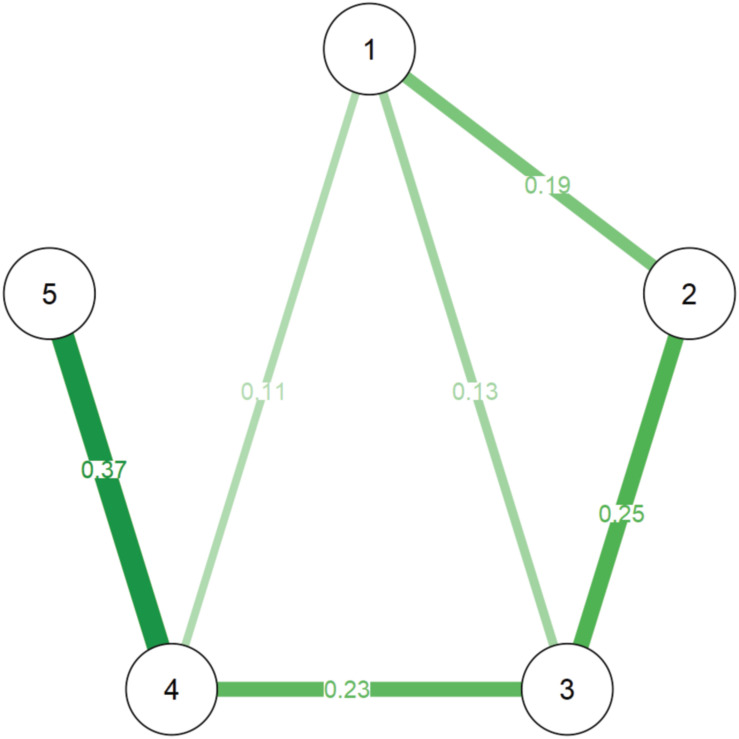
The edges represent regularized partial correlations. Green lines show positive associations. Red lines would have indicated negative ones (none observed). The nodes indicate the variables as it follows: (1) Text Comprehension, (2) Reading fluency, (3) Reading accuracy, (4) Syntactic comprehension, and (5) Non-verbal intelligence.

**FIGURE 2:**
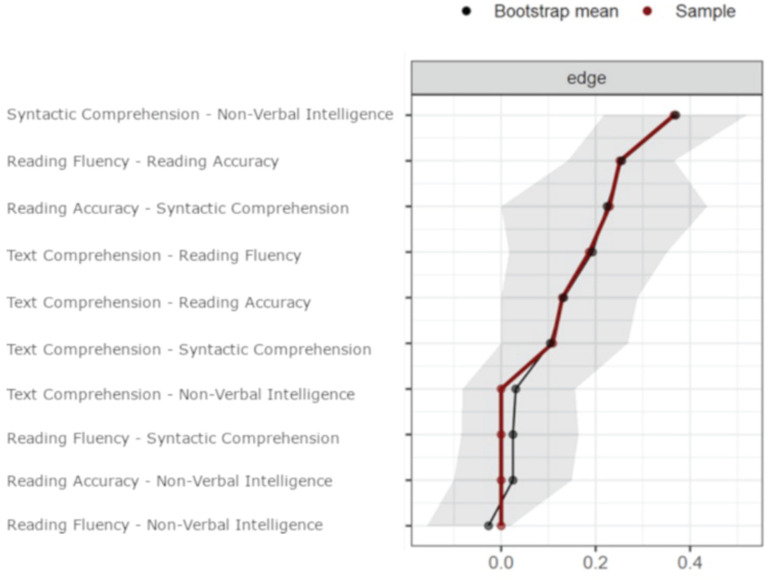
Red dots indicate the edge value of the estimated network. Black dots indicate the average edge value over 1,000 bootstrap resampling. The gray shadow represents the 95% confidence intervals estimated with the bootstrap resampling. Edges are ordered by the estimated strength.

Figure 1 shows the best network estimation representing the relationships among the variables examined. The exact values of all edges, as well as the simple correlations among all variables, are reported in Table 2. The strength centrality index is reported on the diagonal of Table 2. Figure 2 reports the Bootstrap results.

**TABLE 2:** The lower part of the matrix reports the network weights, which correspond to regularized partial correlations (in dark gray). The upper part of the matrix reports simple correlations measured with Pearson’s r (in a light gray background). The diagonal reports the strength centrality index, which is the sum of all the weights that a node receives (in white). Strength also measures the predictability of a node given all the others.

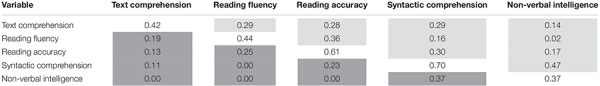

Inspection of the network (Figure 1) suggests a number of main observations:

(1) the comprehension ability on a written text is directly associated with oral syntactic comprehension and text decoding skills;
(2) both reading fluency and reading accuracy are associated with reading comprehension; as network weights are partial correlations, this indicates that both accuracy and fluency have a unique quota of variance shared with reading comprehension;
(3) non-verbal intelligence, as measured with the Raven test, is strongly related to oral syntactic comprehension but not directly with reading comprehension;
(4) reading fluency and reading accuracy are not directly associated with non-verbal intelligence. Inspection of the 95% CI of the edges (obtained with the non-parametric bootstrap analysis; see Figure 2) confirms the reliability of the estimated network. Three observations support this claim:
(a) the edge bootstrap mean overlaps with the estimated edge for all the estimated edges different from 0;
(b) only two estimated edges different from 0 include the 0 in the CI; they are the weakest and 0 stands in the cue of the CI; and
(c) the CI of the estimated edges different from 0 never cross the 0 even when they include it.

## Discussion

This study explored the unique contribution of reading accuracy, reading fluency and linguistic comprehension to the reading comprehension of Italian 3rd-to 5th graders using a network analysis, which is particularly suited to estimating the relations among different but interrelated variables, at the net of each other. Thus, this approach changes the perspective by producing an interpretable output of the simultaneous estimation of each specific association and, could lead to a more comprehensive view of the phenomenon.

Results clearly confirmed the independent and unique contribution of linguistic comprehension over reading comprehension but also pointed out the relevant contribution of both reading fluency and reading accuracy. This pattern of findings is broadly consistent with the predictions of SVR (Gough and Tunmer, 1986; Hoover and Gough, 1990). The decoding measures were not directly associated with non-verbal reasoning, nor was the latter directly associated with reading comprehension; however, it was strongly related to oral syntactic comprehension. In sum, in Italian children, at a stage in which they have already acquired the rudiments of literacy, reading fluency, in terms of reading rate, is a strong and independent predictor of reading comprehension together with reading accuracy and oral comprehension. Notably the identification of this pattern highlights the specific contribution of the network analysis. Simple correlations (see the upper part of Table 2) suggest a less clear pattern where all the variables correlate to some extent with each other, including spurious correlations. The network approach simplifies the picture and removes the non-unique associations, thus unveiling the direct paths.

Our data are only partially consistent with those reported in previous studies of Italian children. First, they confirm that oral comprehension is directly associated with the ability to extract meaning from written texts (see Zamperlin and Carretti, 2010 and Tobia and Bonifacci, 2015, for similar results). Word decoding constitutes the necessary point of entry for reading comprehension. However many of the cognitive operations involved in reading comprehension are shared with the oral language system, thus a set of linguistic and cognitive processes is also essential (see Castles et al., 2018). Reading comprehension, in fact, implies the creation of a mental representation of the passage by combining information from the text to background information, such as sense of the words and their syntactic role in sentences, discernment of grammar rules and also knowledge of things and their relationship. Consistently, in our sample individual ability to understand oral sentences was associated with the ability to derive meaning from written texts.

With regard to reading decoding, our results fit with those reported by Florit and collaborators that reading speed/fluency measures significantly contributed to reading comprehension at least in first graders (Florit et al., 2020) and third graders (Florit et al., 2008); they are at variance with those of other studies which failed to detect any predictive role of reading fluency (Tobia and Bonifacci, 2015).

Various methodological differences may have contributed to this pattern of results. First, the studies included different measures of word recognition, reading comprehension and listening comprehension. As to the different reading measures, Tobia and Bonifacci (2015) and Florit et al. (2020) created two separate latent variables, i.e., one for reading accuracy and the other for reading fluency, based on different accuracy and fluency measures (non-word and word accuracy vs fluency in Florit et al.’s study; text reading accuracy vs fluency in Tobia and Bonifacci’s study) while Roch and Levorato (2009) included only measures of reading accuracy for non-words and reading speed for words. In the present study, we considered reading speed and accuracy in reading a meaningful text (rather than pseudoword decoding), a more functional and ecologically valid task. It seems reasonable that taking into account different reading accuracy and fluency/speed measures should affect the relative load applied by the main constituents of the model. Also, the use of meaningful texts in the present study might have fostered the involvement of semantic components (with children trying to understand the meaning of the passage while reading) and, in turn, yielded a stronger association of reading accuracy with syntactic comprehension as well with text comprehension (with respect to studies that used pseudoword reading as a measure of decoding).

Moreover, there is also evidence that the type of comprehension test and the way in which comprehension is assessed inpacts the evaluation of the SVR: some measures of reading comprehension are more reliant on decoding skills than others (e.g., Cutting and Scarborough, 2006) or touch different aspects of language comprehension (Cain and Oakhill, 2006). For example, short passages, read aloud with cloze/multiple choice questions are more dependent on decoding skills than longer passages, read silently, and with open questions (see for example Keenan et al., 2008). Therefore, open questions rely more on semantic elaboration and the ability to organize the individual response on the basis of most relevant and secondary elements of the text, cloze/multiple choice questions rely more on memory/recognition processes. In the comprehension task used by Tobia and Bonifacci (2015), children were required to read aloud two passages (reading fluency and accuracy were recorded while reading the comprehension text). Texts were of medium length, without figures, and comprehension was tested with open questions that required both text-based comprehension processes (local comprehension) or inferential reasoning (global comprehension). The reading comprehension task paralleled the listening comprehension task; both required text-based and inferential processes. Also, in Zamperlin and Carretti (2010) study, the linguistic comprehension task was a composite measure that included the same passage used to assess reading comprehension but presented in a listening mode. As posited by the authors themselves, the collinearity between measures of reading and linguistic comprehension may have biased the results. In our study, reading decoding parameters and reading comprehension were evaluated with different text passages. The reading comprehension passages were of medium length and comprehension was assessed with multiple-choice questions, which mainly referred to given information. Finally, linguistic comprehension was assessed with an independent task, with respect to reading comprehension, and shared only the multiple-choice response modality with the latter. In synthesis, there were no risks of collinearity between measures and the reading comprehension task adopted relied more on decoding skills than semantic and discourse skills.

Finally, the difference in the results may also be due to the different statistical analyses used in the various studies. In the present research, we use the NA, which is particularly suited for isolating the specific role of each predictor in the SVR model. In fact, simple correlations may depend on a number of potential sources of co-variation, including similarity in the text materials and format. By contrast, the edges in NA indicate relationships that cannot be accounted for by any of the other measures considered. In this way, they return the specific weight of every single variable in the model. Furthermore, NA evaluates the reliability of the observed relationships, thus allowing an estimate of the replicability of results. Based on these considerations, we propose that the present results provide strong evidence that both accuracy and reading rate contribute to the prediction of reading comprehension in Italian children.

Our data support the SVR model also in learners of transparent orthography, specifying that the word recognition component has to contemplate a measure of reading fluency, intended as rate of reading, together with reading accuracy. Findings are consistent with results obtained both in transparent (e.i., Tilstra et al., 2009; Protopapas et al., 2012), intermediate orthographies (such as French, i.e., Massonnié et al., 2019; European Portuguese, i.e., Cadime et al., 2017; Santos et al., 2020) and opaque orthographies (e.g., Joshi and Aaron, 2000; Kershaw and Schatschneider, 2012; Silverman et al., 2013) that support the necessity to add a fluency component to the SVR. After all, fluent reading is the result of a number of processes, that interact each other, and that need be curried out efficiently and automatically (Breznitz, 2006). In other words, fluency captures the development of rapid rates of processing in the various components of reading from letter recognition and orthographic-to-phonological mapping, to word recognition and even semantic encoding (e.g., Wolf and Katzir-Cohen, 2001). Rate is one dimension of automaticity. A process is automatic if it is rapid, undemanding and does not require conscious control or voluntary attention (LaBerge and Samuels, 1974; Logan, 1988, 1997). When applied to reading, these elements indicate parallel, instead of serial, processing of words, they are non-intentional (i.e., the process occurs regardless of the willingness of the reader) and finally they are so quick and smooth that underlying processes are beyond conscious analysis.

The present findings in a transparent orthography seem relevant in a cross-linguistic perspective. On one hand, children learning a transparent orthography such as Italian rely more on small grain sizes in reading with respect to children learning more opaque orthographies that use a larger grain size (e.g., Marinelli et al., 2014, 2016). This makes reading in transparent orthographies a more serial process, well grasped by the reading rate dimension. On the other hand, the higher accuracy reached by Italian children after only a few years of schooling, as well as the smaller inter-individual variability, could have made the accuracy measure less sensitive to capturing word reading proficiency and in turn less related to reading comprehension with respect to more opaque orthographies. Note that, also in opaque orthographies, to explain some inconsistency among studies, it was proposed that once children become more accurate in their word reading fluency could be a more sensitive indicator of word reading ability and variability in fluency effectively accounts for reading comprehension (Language and Reading Research Consortium, 2015). Nevertheless, we found an independent, strong and unique contribution of both reading fluency and accuracy in explaining reading comprehension. Thus, the hypothesis (e.g., Müller and Brady, 2001) of a weaker relationship between decoding and reading comprehension, and conversely a stronger one between linguistic and reading comprehension among learners of consistent respect to opaque orthographies was not confirmed.

The present study has a number of limitations. We examined children in the final years of primary school, i.e., when the basic assets of reading are acquired and lexical reading is detectable also in a language with a highly transparent orthography such as Italian (Burani et al., 2002). While there is some indication that children are relatively homogeneous in the 8–10 year age range, it would have been interesting to detail the developmental trend over the three classes examined. However, this proved difficult for methodological reasons. First, the sample size for this study would have been reasonable for network analysis if the three classes had been collapsed together. If we had split it into three sub-groups, each one would have been too small to allow for reliable estimates. Second, if we had added age as a node it would have been technically feasible but incorrect as ages are not independent of grade level. Thus, including a continuous variable in the model would actually have hidden an ordinal variable (with three levels), and the GGM is poor in estimating ordinal variables with only a few levels. Finally, we analyzed z-scores in order to correct for differences in age and materials used. Thus, adding an age node or splitting graphs in three would provided a sort of double-dipping in the age variable, which is somehow under control when proper materials and standardizations are used. Thus, even though the general confounds related to age were under control, we have to conclude that it was impossible to detail the developmental trend within the age span considered, which is a limitation of the study. Further work is needed to clarify this point.

Another limitation concerns the possibility of generalizing the present results. We aimed to examine a sample without imposing limits of performance in the critical variables and only used low non-verbal intelligence as an exclusion criterion. Further, we obtained our sample from middle-class areas without critical migratory flows. Results confirmed the presence of great variability across children in all critical variables, including reading accuracy, which is a measure at risk for ceiling effects in a highly transparent orthography such as Italian. However, although we attempted to limit as much as possible potential selection bias (e.g., only children with very low non-verbal intelligence were excluded), our sample does not include a stratification of demographic variables. Thus, it would be incorrect to consider our sample as perfectly representative of the entire Italian primary school population, and generalizing results should be undertaken with caution.

Finally, our results have educational and rehabilitative implications. Thus, even when decoding deficits manifest as inadequate reading fluency they are expected to have an indirect but important influence on reading comprehension. Decoding and comprehension are likely to proceed well when both processes operate “automatically,” which also means at a reasonable rate. In this vein, it was found that reading rate contributes to the understanding of reading passages because it has a mediating role between reading strategy awareness/use and reading comprehension (Rahimi and Babaei, 2021). Consequently, in the case of poor comprehenders the assessment of decoding skills might help in choosing the appropriate rehabilitation interventions. Moreover, reading trainings fostering faster and parallel word recognition will have carry-over effects on reading comprehension. In a recent study on English as a foreign language, it was found that a reading training that significantly improving students’ reading rate also had a significant role in empowering their ability to process and better grasp the text (Rahimi and Babaei, 2021).

Overall, our data support the SVR model of reading also in learners of a transparent orthography. Furthermore, they indicate that when reading rate is taken as a component of reading fluency it effectively captures the dimension of automaticity and should be taken into account together with reading accuracy and the processes involved in linguistic comprehension in predicting text comprehension outcomes.

## Data Availability Statement

The raw data supporting the conclusions of this article will be made available by the authors, without undue reservation.

## Ethics Statement

The studies involving human participants were reviewed and approved by Ethics Committee of Psychological Research of the Department of History, Society and Human Studies—University of Salento (Prot. 101206—29th July 2020). Written informed consent to participate in this study was provided by the participants’ legal guardian/next of kin.

## Author Contributions

PA, CM, and PZ contributed to the conception and design of the study. LM organized the database. DR organized the database and performed the statistical analysis. PA wrote the first draft of the manuscript. All authors wrote sections of the manuscript, contributed to manuscript revision, read, and approved the submitted version.

## Conflict of Interest

The authors declare that the research was conducted in the absence of any commercial or financial relationships that could be construed as a potential conflict of interest.

## Publisher’s Note

All claims expressed in this article are solely those of the authors and do not necessarily represent those of their affiliated organizations, or those of the publisher, the editors and the reviewers. Any product that may be evaluated in this article, or claim that may be made by its manufacturer, is not guaranteed or endorsed by the publisher.

## Footnotes

1 Reading fluency is often measured as a combination of rate and accuracy: the number of correct words read aloud in 1 min (e.g., Shinn et al., 1992; Fuchs et al., 2001).

2 In these studies fluency is measured in terms of syllables per second (s/s); thus, it captures the dimension of reading rate.
